# Plant beneficial traits of endophytic bacteria associated with fennel (*Foeniculum vulgare* Mill.)

**DOI:** 10.3934/microbiol.2024022

**Published:** 2024-06-18

**Authors:** Vyacheslav Shurigin, Li Li, Burak Alaylar, Dilfuza Egamberdieva, Yong-Hong Liu, Wen-Jun Li

**Affiliations:** 1 State Key Laboratory of Desert and Oasis Ecology, Xinjiang Key Laboratory of Biodiversity Conservation and Application in Arid Lands, Xinjiang Institute of Ecology and Geography, Chinese Academy of Sciences, Urumqi 830011, Xinjiang, China; 2 Department of Molecular Biology and Genetics, Faculty of Arts and Sciences, Agri Ibrahim Cecen University, Agri 04100, Turkey; 3 Institute of Fundamental and Applied Research, National Research University TIIAME, Tashkent 100000, Uzbekistan; 4 Faculty of Biology, National University of Uzbekistan, Tashkent 100174, Uzbekistan; 5 State Key Laboratory of Biocontrol and Guangdong Provincial Key Laboratory of Plant Stress Biology, Sun Yat-Sen University, Guangzhou, 510275, China

**Keywords:** medicinal plant, plant beneficial, antagonism, endophytes

## Abstract

In this study, we used 16S rRNA gene sequence analysis to describe the diversity of cultivable endophytic bacteria associated with fennel (*Foeniculum vulgare* Mill.) and determined their plant-beneficial traits. The bacterial isolates from the roots of fennel belonged to four phyla: *Firmicutes* (BRN1 and BRN3), *Proteobacteria* (BRN5, BRN6, and BRN7), *Gammaproteobacteria* (BRN2), and *Actinobacteria* (BRN4). The bacterial isolates from the shoot of fennel represented the phyla *Proteobacteria* (BSN1, BSN2, BSN3, BSN5, BSN6, BSN7, and BSN8), *Firmicutes* (BSN4, BRN1, and BRN3), and *Actinobacteria* (BRN4). The bacterial species *Bacillus megaterium*, *Bacillus aryabhattai*, and *Brevibacterium frigoritolerans* were found both in the roots and shoots of fennel. The bacterial isolates were found to produce siderophores, HCN, and indole-3-acetic acid (IAA), as well as hydrolytic enzymes such as chitinase, protease, glucanase, and lipase. Seven bacterial isolates showed antagonistic activity against *Fusarium culmorum*, *Fusarium solani*, and *Rhizoctonia. solani*. Our findings show that medicinal plants with antibacterial activity may serve as a source for the selection of microorganisms that exhibit antagonistic activity against plant fungal infections and may be considered as a viable option for the management of fungal diseases. They can also serve as an active part of biopreparation, improving plant growth.

## 1. Introduction

Fennel (*Foeniculum vulgare* Mill.) is an annual herbaceous plant belonging to the family *Umbelliferae* (*Apiaceae*) and cultivated in many countries [1]. Fennel's fruits contain highly valuable volatiles and fatty oils, which are used in the food industry, cosmetics, and medicine [2]. Moreover, fennel exhibits antioxidant [3], antimicrobial [4]–[6], anti-inflammatory [7], antithrombotic [8], antidiabetic [9], cytoprotection antitumor [10], anti-diarrheic, and anti-spasmodic activities [11].

Fennel is commercially cultivated in many countries; however, this crop is attacked by several fungal diseases such as collar rot (*Sclerotium rolfsii*), damping off and root rot (*Pythium* spp.), vascular wilt (*Fusarium oxysporum*), root and foot rot (*Rhizoctonia solani*) [12], brown rot and wilt (*Phytophthora megasperma)* [13], stem rot (*Sclerotinia sclerotiorum*) [14], and blight and leaf spot (*Alternaria alternata*) [15].

Production of fennel through eco-friendly technology is an important approach, ensuring organic fennel. The application of plant-beneficial microbes is considered as an alternative eco-friendly approach to improving medicinal plant health [16]–[18]. Among these microbes, endophytic bacteria that colonize plant internal tissues, roots, leaves, and stems can provide beneficial effects to plants [19]–[21]. There are many reports on the diversity of endophytic bacteria associated with medicinal plants, and their biological activity has been reported, e.g., *Ziziphora capitata*, *Hypericum perforatum* [16], *Aloe vera*, [22], and *Origanum vulgare* [23]. Endophytes colonizing plant tissue are assumed to play an important role in the synthesis of biologically active compounds and also protect plants from soil-borne disease [24]–[26]. Several mechanisms underlying plant beneficial effects have been reported, including the production of phytohormones, cell wall–degrading enzymes, hydrogen cyanide (HCN), and ACC deaminase [27],[28]. Moreover, there is evidence that the chemical composition of the exudate affects the microbial diversity and activity associated with plants [29]. For example, bacteria associated with medicinal plants such as *Matricaria chamomilla*, *Baccharoides anthelmintica*, and *Calendula officinalis* exhibit antimicrobial activity similar to that of the host plant [30]–[32].

To date, there have been only a few reports of endophytes associated with fennel and their beneficial effects on plants, despite numerous studies reporting on the phytochemical contents and biological activity of fennel (*Foeniculum vulgare* Mill.). To enhance our understanding of the function of endophytes in plant growth and development, it is crucial to gain knowledge about the physiological activities of endophytic bacteria associated with medicinal plants. In the current study, we aim (1) to isolate and identify culturable endophytic bacteria associated with fennel by using 16S rRNA gene analysis, and (2) to evaluate their plant-beneficial properties.

## 2. Materials and methods

### 2.1. Plant sample collection

In June 2019, fennel (*Foeniculum vulgare* Mill.) was harvested from Ugam-Chatkal State Biosphere Reserve, Uzbekistan (41°15′27.7″N, 69°54′41.4″E), a remote and forested region situated in the Western Tien Shan province. Ten individual plants with their root systems were collected using sterile gloves at a distance of 12–15 m. They were then stored in zip-lock plastic bags and brought to the lab for additional analysis.

### 2.2. Isolation of endophytic bacteria

For sterilization of plant roots and leaves, 10% NaClO and 70% ethanol were used. Then, they were rinsed in 2 L of sterile water (2 min) five times. The root and leaves (10 g each) were squeezed out with a sterile mortar and mixed with 90 mL of phosphate buffer solution [33]. The mixtures resulting from dilutions (10^1^–10^5^) were spread out in 100 µL of tryptic soy agar (TSA) (BD, Difco Laboratories, USA) with an addition of 50 µg/mL of nystatin and stored in a thermostat for 96 h at 28 °C. Every single colony that had a distinct color, shape, surface, and consistency was the source of the new isolates, and the plates were examined for bacterial growth.

### 2.3. Identification of bacteria

The heat treatment method was used to isolate bacterial DNA [34] as follows: The bacterial isolates were cultivated on Petri plates with TSA at 28 °C for 72 h. Subsequently, the colonies were transferred into Eppendorf tubes with 300 µL of sterile Milli-Q water, incubated at 90 °C for 20 min in a dry block heater (IKA Works, Inc., Wilmington, USA), and centrifuged at 12,000 rpm for 5 min. The presence of DNA in the tubes was tested using gel electrophoresis and quantified with NanoDrop™ One (Thermo Fisher Scientific Inc., Waltham, USA).

The 16S rRNA gene sequences were amplified from the isolated DNA during polymerase chain reaction (PCR) using the following primers: 27F 5′-GAGTTTGATCCTGGCTCAG-3′ (Sigma-Aldrich, St. Louis, Missouri, USA) and 1492R 5′-GAAAGGAGGTGATCCAGCC-3′ (Sigma-Aldrich, St. Louis, Missouri, USA) [35]. The bacterial isolates were differentiated using restriction fragment length polymorphism (RFLP) analysis of the obtained 16S rRNA gene products, as described by Jinneman et al. [36]. The digested DNA fragments were examined using gel electrophoresis (1% agarose gel). The gel was visualized using a digital gel imaging system (Gel-Doc XR TM+, Bio-Rad Laboratories, USA). Identical isolates were eliminated, and the rest were sequenced. The ABI PRISM BigDye 3.1 Terminator Cycle Sequencing Ready Reaction Kit (Applied Biosystems) was used for the sequencing of PCR products. The Chromas (v. 2.6.5) and EMBOSS Explorer (http://emboss.bioinformatics.nl/) software were used for the evaluation, correction, and alignment of the nucleotide sequences.

The 16S rRNA gene sequences were checked for identity with the relative sequences from the GenBank of NCBI (http://www.ncbi.nlm.nih.gov/) using the Basic Local Alignment Search Tool (BLAST). The Clustal Omega (https://www.ebi.ac.uk/Tools/msa/clustalo/) online software was used for multiple alignments of all obtained and relative 16S rRNA gene sequences. The maximum composite likelihood method [37] was used for counting the evolutionary distances. The percentage of replicate trees in which the associated taxa clustered together in the bootstrap test (500 replicates) are shown next to the branches of a phylogenetic tree, which was built using MEGA X software [38].

Upon the deposition of the obtained 16S rRNA gene sequences to GenBank, they were assigned the following accession numbers: MT310821-MT310835.

### 2.4. Antifungal activity of endophytes

The ability of cell-free solutions of endophytic bacterial isolates and plant extracts to inhibit plant pathogenic fungi *Rhizoctonia solani*, *Fusarium culmorum* (Wm.G.Sm.) Sacc., and *F. solani* (Mart.) Sacc. J.G. Kühn was investigated in the way detailed by Egamberdieva et al. [39].

The bacterial isolates were grown in TSB broth for three days, and 50 µL of bacterial cultures were dropped into a hole in PDA plates (4 mm in diameter). Fungal strains were obtained from the culture collection of microorganisms at the National University of Uzbekistan, and they were grown in PDA plates at 28 °C for five days. Disks of fresh fungus cultures (5 mm in diameter) were cut out and placed 2 cm away from the hole filled with bacterial filtrate. The plates were sealed with Parafilm®M and incubated at 28 °C in darkness until the fungi had grown over the control plates without bacteria. Antifungal activity was recorded as the width of the growth-inhibition zone between the fungus and the test bacterium.

### 2.5. Plant-beneficial traits of endophytes

On TSA media, the ability of bacterial isolates to produce hydrogen cyanide (HCN) was examined. The color change of filter paper immersed in a 1% picric acid and 2% sodium carbonate solution and put on Petri plates was measured [40]. The bacterial isolates' ability to produce siderophores was determined using the following method described by Schwyn and Neilands [41]. Protease secretion was revealed by growing strains on TSA plates (20 times diluted) amended with skimmed milk to a final concentration of 5%. The halo appearing on the first to the second day of cultivation around colonies indicated the presence of extracellular protease [42]. Furthermore, β-1,3 and β-1,4glucanase activity was tested using the substrate lichenan (Sigma-Aldrich, St. Louis, MO) in top agar plates (Walsh et al. 1995). The production of chitinase by bacterial isolates was determined on colloidal chitin medium using the Malleswari and Bagyanarayana [44] method. The lipase activity of bacterial strains was determined by the Tween lipase indicator assay. Bacterial strains were grown in LC agar (LB agar containing 10 mM MgSO_4_ and 5 mM CaCI_2_) containing 2% Tween 80 at 28 °C [45]. After five days, the degradation of Tween was taken as a clear halo around the bacterial inoculum. Using the technique outlined by Bano and Musarrat [46], the synthesis of IAA (indole 3-acetic acid) by endophytic isolates was investigated. The IAA concentration in culture was calculated by using a calibration curve of pure IAA as a standard (Sigma-Aldrich, Merck). According to Egamberdieva and Kucharova's description [47], ACC deaminase synthesis was investigated with 1-aminociclopropane-1-carboxylacid (ACC) as the only N source. The P-solubilization ability of bacterial isolates was performed as previously described by Chen et al. [48].

### 2.6. Plant growth promotion

After being cultured for 72 h in tryptic soy broth (TSB; Sigma-Aldrich), the bacterial cultures were adjusted to an optical density of 0.1 (OD_620_ = 0.1) at 620 nm, which corresponds to approximately 10^8^ cells/mL. The fennel seeds were dipped into bacterial solutions and, after 5 min, inoculated seeds with bacteria were sown in pots (two seeds per pot) (12 cm in diameter and 16 cm in depth) filled with 500 g of soil. After germination, one seedling was kept per pot. In the experiment, a randomized design was employed, with each treatment consisting of 10 pots. There were two treatments in the pot experiment: pots with the plant uninoculated with bacteria and pots with plants inoculated with bacteria. The plants were grown for two weeks at 24–26 °C during the day and 17–18 °C at night, with 40% humidity. The shoot and root lengths as well as the dry weight were measured [47].

### 2.7. Statistical analyses

Using Microsoft Excel 2010's analysis of variance software, the data were examined for statistical significance. Data obtained from the plant growth test were subjected to analysis of variance (ANOVA) with SPSS software (version 15) at p < 0.05. The results are presented as average means and standard error (SE). The difference between means was compared by a high-range statistical domain (HSD) using Tukey's test. The treatment means were separated by the least significant difference (LSD) test at p < 0.05.

## 3. Results

### 3.1. Isolation and identification of cultivable endophytic bacteria

**Figure 1.**
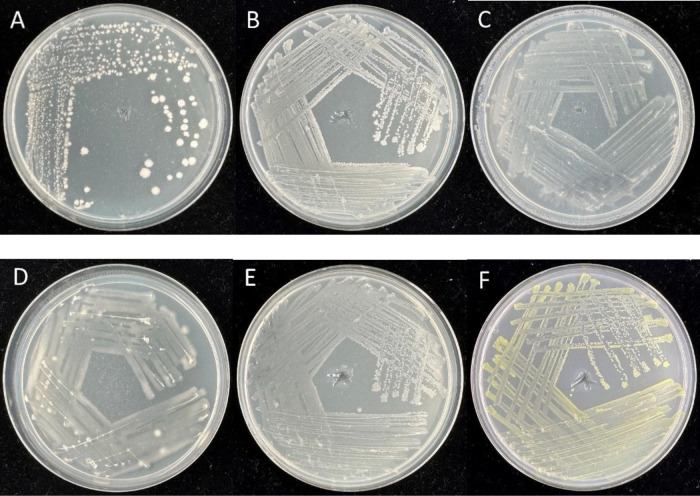
Colonies of some isolated bacteria. A. BRN3. B. BRN1. C. BRN6. D. BRN7. E. BRN2. F. BSN6.

In total, 60 bacterial isolates were obtained from the plant tissues of fennel. The RFLP analysis was utilized for the selection of similar isolates. After RFLP analysis, 18 bacterial isolates were selected (7 from roots and 11 from shoots) and siblings were removed. The colonies of some isolates with plant-beneficial traits are shown in Figure 1.

**Figure 2.**
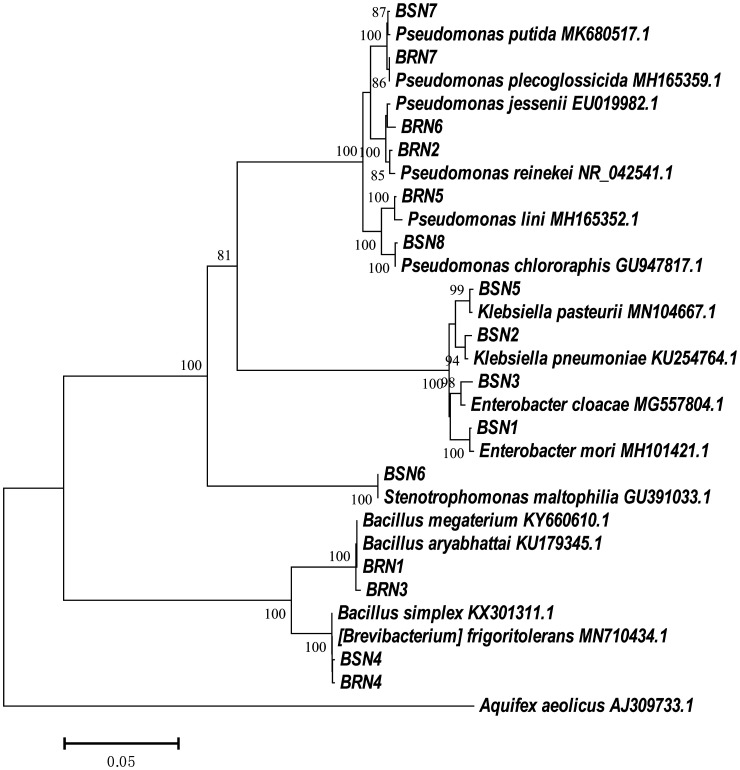
Neighbor-joining phylogenetic tree based on 16S rRNA gene sequences isolated from endophytic bacteria of fennel (*Foeniculum vulgare* Mill.), showing the relationship of isolated strains to their closest relatives in GenBank.

All isolates were determined using the BLAST basic local alignment search tool and matched with correlative strains from the NCBI GenBank. The isolates were 98.95%–99.93% identical to their closest relatives registered in GenBank®. Sequence similarities of endophyte bacteria isolated from the root and shoot systems of fennel are given in Tables 1 and 2. The length of the identified nucleotide sequences of 16S rRNA gene in the isolates varied from 1408 to 1470 bp and was noted as adequate for confidential identification based on 16S rRNA gene analysis using the BLAST tool. All isolated strains got their accession numbers (Tables 1 and 2). As shown in Table 1, the roots of fennel harbored seven species belonging to four phyla: *Firmicutes* (BRN1 and BRN3), *Proteobacteria* (BRN5, BRN6, and BRN7), *Gammaproteobacteria* (BRN2), and *Actinobacteria* (BRN4). Table 2 comprises 11 strains isolated from shoots of fennel and represents the phyla *Proteobacteria* (BSN1, BSN2, BSN3, BSN5, BSN6, BSN7, and BSN8), *Firmicutes* (BSN4, BRN1, and BRN3) and *Actinobacteria* (BRN4) (Figure 2). Above all, *Bacillus megaterium*, *Bacillus aryabhattai*, and *Brevibacterium frigoritolerans* were found both in the roots and shoots of fennel.

**Table 1.** Sequence similarities of endophyte bacteria isolated from the root system of fennel (*Foeniculum vulgare* Mill.) with sequences registered in GenBank.

Isolated strains deposited to GenBank			Closest match (16S ribosomal RNA genes) (GenBank)		

Strain	Length (bp)	Accession number	Reference strains	Accession number	Percent identity, %
BRN1	1457	MT310821	*Bacillus megaterium*	KY660610.1	99.93
BRN2	1408	MT310822	*Pseudomonas reinekei*	NR_042541.1	99.50
BRN3	1454	MT310823	*Bacillus aryabhattai*	KU179345.1	99.79
BRN4	1459	MT310824	*[Brevibacterium] frigoritolerans*	MN710434.1	99.79
BRN5	1450	MT310825	*Pseudomonas lini*	MH165352.1	99.24
BRN6	1421	MT310826	*Pseudomonas jessenii*	EU019982.1	99.43
BRN7	1444	MT310827	*Pseudomonas plecoglossicida*	MH165359.1	99.93

**Table 2.** Sequence similarities of endophyte bacteria isolated from shoots of fennel (*Foeniculum vulgare* Mill.) with sequences registered in GenBank.

Isolated strains deposited to GenBank			Closest match (16S ribosomal RNA genes) (GenBank)		

Strain	Length (bp)	Accession number	Reference strains	Accession number	Percent identity, %
BSN1	1439	MT310828	*Enterobacter mori*	MH101421.1	99.31
BSN2	1438	MT310829	*Klebsiella pneumoniae*	KU254764.1	99.24
BSN3	1428	MT310830	*Enterobacter cloacae*	MG557804.1	98.95
BSN4	1470	MT310831	*Bacillus simplex*	KX301311.1	99.59
BSN5	1443	MT310832	*Klebsiella pasteurii*	MN104667.1	99.17
BSN6	1431	MT310833	*Stenotrophomonas maltophilia*	GU391033.1	99.93
BSN7	1448	MT310834	*Pseudomonas putida*	MK680517.1	99.65
BSN8	1444	MT310835	*Pseudomonas chlororaphis*	GU947817.1	99.79
BRN1	1455	MT310821	*Bacillus megaterium*	KY660610.1	99.66
BRN3	1463	MT310823	*Bacillus aryabhattai*	KU179345.1	99.73
BRN4	1458	MT310824	*[Brevibacterium] frigoritolerans*	MN710434.1	99.73

### 3.2. Antifungal activity of endophytic bacteria

**Figure 3.**
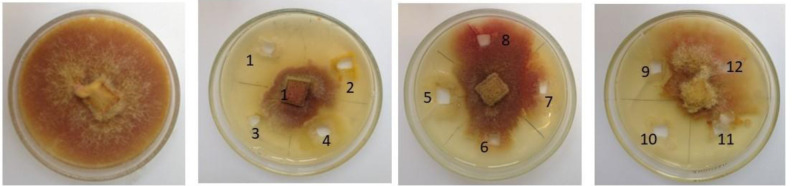
Antagonistic activity of bacterial strains against the plant pathogenic fungi *Fusarium culmorum*. 1. *P. reinekei* BRN2; 2. *B. megaterium* BRN1; 3. *P. putida* BSN7; 4. *B. aryabhattai* BRN3; 5. *P. plecoglossicida* BRN7; 6. *E. mori* BSN1; 7. *K. pneumoniae* BSN2; 8. *E. cloacae* BSN3; 9. *B. simplex* BSN4; 10. *P. jessenii* BRN6; 11. *B. frigoritolerans* BRN4; 12. *K. pasteurii* BSN5.

The antifungal activity of the isolated endophytic bacteria was evaluated using three plant pathogenic fungi: *F. culmorum*, *F. solani*, and *R. solani* (Table 3, Figure 3). Among all tested endophytic bacteria, *P. reinekei* BRN2, *P. jessenii* BRN6, *S. maltophilia* BSN6, and *P. chlororaphis* BSN8 exhibited strong inhibition against three tested plant pathogenic fungi. *P. lini* BRN5, *P. plecoglossicida* BRN7, and *B. simplex* BSN4 showed antifungal activity against two tested fungal plant pathogens: *F. culmorum* and *F. solani*. *B. megaterium* BRN1 and *B. aryabhattai* BRN3 demonstrated antifungal activity against only one fungus *R. solani*.

**Table 3.** Antifungal activity of bacterial endophytes from fennel (*Foeniculum vulgare* Mill.) against plant pathogenic fungi.

Treatments	Inhibition zone in diameter (mm)		
	*F. culmorum* (Wm.G.Sm.) Sacc.	*F. solani* (Mart.) Sacc.	*R. solani* J.G. Kühn
*Bacillus megaterium* BRN1	-	-	5 ± 1
*Pseudomonas reinekei* BRN2	8 ± 1	7 ± 1	11 ± 1
*Bacillus aryabhattai* BRN3	-	-	6 ± 1
*[Brevibacterium] frigoritolerans* BRN4	-	-	-
*Pseudomonas lini* BRN5	7 ± 1	6 ± 1	-
*Pseudomonas jessenii* BRN6	10 ± 1	10 ± 1	13 ± 1
*Pseudomonas plecoglossicida* BRN7	5 ± 1	4 ± 1	-
*Enterobacter mori* BSN1	-	-	-
*Klebsiella pneumoniae* BSN2	-	-	-
*Enterobacter cloacae* BSN3	-	-	-
*Bacillus simplex* BSN4	5 ± 1	6 ± 1	-
*Klebsiella pasteurii* BSN5	-	-	-
*Stenotrophomonas maltophilia* BSN6	7 ± 1	7 ± 1	9 ± 1
*Pseudomonas putida* BSN7	-	-	7 ± 1
*Pseudomonas chlororaphis* BSN8	10 ± 1	9 ± 1	11 ± 1
Plant extract	4 ± 1	3 ± 1	4 ± 1

*“-“ no formation of inhibition zone*

### 3.3. Plant growth–promoting activity of endophytic bacteria

The isolated endophytes were tested for their ability to stimulate the growth of fennel seedlings (Table 4). Some of the tested bacteria showed high plant-growth promotion in fennel seedlings. Seed inoculation with strain *B. aryabhattai* BRN3 resulted in a 21.5% and 24.5% increase in shoot and root length, respectively as compared to the control. The shoot and root dry mass also rose to 25.2% and 24.6%, respectively, as compared to the control. The strains *B. megaterium* BRN1, *P. reinekei* BRN2, *P. jessenii* BRN6, *P. plecoglossicida* BRN7, *K. pneumoniae* BSN2, and *S. maltophilia* BSN6 were less effective, and increased shoot length up to 7.6%–17.7% and root length up to 6.1%–18.4% in comparison with control. The strains *P. chlororaphis* BSN8, *P. lini* BRN5, *B. simplex* BSN4, *K. pasteurii* BSN5, *P. putida* BSN7, and *B. frigoritolerans* BRN4 did not exhibit, or showed very low, plant growth–promoting activity. Two strains (*E. mori* BSN1 and *E. cloacae* BSN3) inhibited the growth of fennel seedlings and reduced shoot and root length and dry weight.

**Table 4.** Length and dry weight of shoot and root of fennel (*Foeniculum vulgare* Mill.) when seeds were inoculated with endophytic bacteria. Plants were grown in pots for two weeks.

Treatment	Shoot length (cm)	Root length (cm)	Shoot dry weight (g)	Root dry weight (g)
Control	7.9 ± 0.65bc	9.8 ± 0.67bc	1.43 ± 0.04bc	0.434 ± 0.01bc
*B. megaterium* BRN1	9.0 ± 0.81a	11.1 ± 0.83ab	1.65 ± 0.07ab	0.495 ± 0.01ab
*P. reinekei* BRN2	8.7 ± 0.91ab	10.8 ± 0.66ab	1.58 ± 0.07ab	0.473 ± 0.02ab
*B. aryabhattai* BRN3	9.6 ± 0.72 a	12.2 ± 0.82a	1.79 ± 0.06a	0.541 ± 0.01a
*B. frigoritolerans* BRN4	7.9 ± 0.60bc	9.8 ± 0.77bc	1.43 ± 0.08bc	0.434 ± 0.02bc
*P. lini* BRN5	8.1 ± 0.72bc	9.8 ± 0.92bc	1.47 ± 0.06bc	0.437 ± 0.01bc
*P. jessenii* BRN6	9.3 ± 0.61a	11.6 ± 0.78a	1.71 ± 0.08a	0.516 ± 0.01a
*P. plecoglossicida* BRN7	8.6 ± 0.92ab	10.5 ± 0.56b	1.61 ± 0.05ab	0.459 ± 0.02b
*E. mori* BSN1	7.7 ± 0.55c	9.5 ± 0.88c	1.39 ± 0.08c	0.427 ± 0.01c
*K. pneumoniae* BSN2	8.5 ± 0.77b	10.7 ± 0.93ab	1.53 ± 0.08b	0.466 ± 0.03ab
*E. cloacae* BSN3	7.5 ± 0.66c	9.3 ± 0.78c	1.34 ± 0.07c	0.422 ± 0.01c
*B. simplex* BSN4	8.2 ± 0.51bc	9.8 ± 0.81bc	1.48 ± 0.06bc	0.434 ± 0.01bc
*K. pasteurii* BSN5	8.0 ± 0.56bc	9.8 ± 0.78bc	1.46 ± 0.09bc	0.435 ± 0.02bc
*S. maltophilia* BSN6	8.7 ± 0.62ab	10.4 ± 0.94b	1.56 ± 0.04ab	0.451 ± 0.02ab
*P. putida* BSN7	8.3 ± 0.59b	10.2 ± 0.88bc	1.50 ± 0.04b	0.445 ± 0.01b
*P. chlororaphis* BSN8	8.2 ± 0.70bc	10.0 ± 0.76bc	1.48 ± 0.04bc	0.441 ± 0.02b

*different letters indicate significant differences based on Turkey's HSD test at p < 0.05.

### 3.4. Plant-beneficial traits

Detailed results of plant-beneficial traits of endophytic bacteria isolated from fennel (*Foeniculum vulgare* Mill.) are given in Table 5. According to the results, *B. megaterium* BRN1, *P. reinekei* BRN2, *B. aryabhattai* BRN3, *P. jessenii* BRN6, *P. plecoglossicida* BRN7, *K. pneumoniae* BSN2, *S. maltophilia* BSN6, and *P. putida* BSN7 produced IAA. Among these bacterial strains, the highest IAA synthesis was demonstrated in the root- and shoot-associated bacteria *B. aryabhattai* BRN3. Siderophore production was observed in 8 out of 15 bacterial isolates. Seven isolates out of 15 showed ACC deaminase production and phosphate solubilization. Nine of the strains also showed hydrogen cyanide (HCN) production. The strains were also tested for fungi cell wall–degrading enzymes (chitinase, glucanase, protease, and lipase) production. It was revealed that strains *S. maltophilia* BSN6, *P. jessenii* BRN6, *B. simplex* BSN4, and *P. reinekei* BRN2 produced three out of four tested enzymes. The strains *P. chlororaphis* BSN8, *P. plecoglossicida* BRN7, *P. putida* BSN7, and *P. lini* BRN5 showed production of two enzymes. The strains *E. cloacae* BSN3, *K. pneumoniae* BSN2, and *B. aryabhattai* BRN3 produced only one of the tested enzymes. The strains *K. pasteurii* BSN5, *B. frigoritolerans* BRN4, *E. mori* BSN1, and *B. megaterium* BRN1 did not produce any of the tested enzymes.

**Table 5.** Plant-beneficial traits of endophytic bacteria isolated from fennel (*Foeniculum vulgare* Mill.).

Bacterial strains		Siderophores	PSB	HCN	IAA (µg/mL)	ACC deaminase	Cell wall–degrading enzymes			
							Chitinase	Glucanase	Protease	Lipase
*B. megaterium* BRN1	RS	-	+	+	6.3 ± 0.3	+	-	-	-	-
*P. reinekei* BRN2	R	+	-	+	5.5 ± 0.3	-	+	+	+	-
*B. aryabhattai* BRN3	RS	-	+	-	7.8 ± 0.3	+	-	-	+	-
*B. frigoritolerans* BRN4	RS	-	-	-	0	-	-	-	-	-
*P. lini* BRN5	R	+	-	+	0	+	-	+	+	-
*P. jessenii* BRN6	R	+	+	+	6.8 ± 0.3	-	+	+	-	+
*P. plecoglossicida* BRN7	R	+	-	+	4.5 ± 0.2	-	-	+	+	-
*E. mori* BSN1	S	-	-	-	0	+	-	-	-	-
*K. pneumoniae* BSN2	S	-	-	-	5.6 ± 0.3	-	-	-	+	-
*E. cloacae* BSN3	S	-	-	-	0	-	-	-	-	+
*B. simplex* BSN4	S	+	+	+	0	-	-	+	+	+
*K. pasteurii* BSN5	S	-	-	-	0	+	-	-	-	-
*S. maltophilia* BSN6	S	+	+	+	4.3 ± 0.3	-	+	-	+	+
*P. putida* BSN7	S	+	+	+	2.8 ± 0.3	+	+	-	-	+
*P. chlororaphis* BSN8	S	+	+	+	-	+	-	+	+	-

*“+” positive, “-“ negative”, R: bacteria isolated from root; S: bacteria isolated from shoot*.

## 4. Discussion

Plant-associated endophytic bacteria are vital to the health of plants. They have been thought to be a valuable source of physiologically active chemicals because they create a variety of beneficial metabolites [19],[27],[49]. Furthermore, some genetic explanations for the endophytic lifestyle of this bacterium have been offered by the whole-genome gene content study of plant-associated bacteria. The gene content analysis identified genes involved in motility, biofilm production, siderophore biosynthesis, chemotaxis, and osmoprotectant production, indicating their potential benefit for plant performance [50],[51].

This research is the first analysis of endophytic bacteria found in fennel (*Foeniculum vulgare* Mill.) growing in the desert Ugam-Chatkal State Biosphere Reserve in Uzbekistan. Profiling of endophytic bacteria isolated from the roots and shoots of fennel demonstrated that these included 18 isolates belonging to the genera *Bacillus* (5), *Pseudomonas* (6), *Brevibacterium* (2), *Enterobacter* (2), *Klebsiella* (2), and *Stenotrophomonas* (1). Similar bacterial species were isolated from the tissues of other medicinal plants, e.g., *Bacillus megaterium* from *Lonicera japonica* [52], *Enterobacter cloacae* from *Tridax procumbens* Linn. [53], *Bacillus aryabhattai* from *Pterocarpus santalinus* [54], *Brevibacterium frigoritolerans* from *Ferula songorica* [55], or *Stenotrophomonas maltophilia* from *Armoracia rusticana* [20]. Notably, we observed *Bacillus megaterium*, *Bacillus aryabhattai*, and [*Brevibacterium*] *frigoritolerans* both in the roots and the shoots of fennel, which can be the result of the chemotactic movement of bacteria toward plant roots in response to exudates released by the plant. [56]. The number of diverse isolates from shoots was higher than from roots. However, the diversity of culturable bacteria in plants represents only a fraction of the total microbial diversity. Advanced techniques like metagenomics and high-throughput sequencing are essential to capture a more comprehensive picture of the microbial communities associated with plants. Our study focused on the plant-beneficial traits of culturable bacteria associated with plants. Shi et al. [57] studied the total microbial community in potato tissues using Illumina MiSeq sequencing and found a higher diversity of bacteria species in roots than in shoots. The higher microbial diversity in roots compared to shoots is a result of the nutrient-rich environment, direct soil contact, favorable microenvironmental conditions, symbiotic relationships, and constant exposure to a diverse soil microbiome.

Endophytes support plant health by enhancing nutrient acquisition, promoting growth, suppressing diseases, increasing abiotic stress tolerance, and providing disease control. They exhibit several traits that help plants thrive. In our study, several bacterial endophytes showed antagonistic action against the plant pathogenic fungi *F. oxysporum, F. solani*, and *R. solani*. The antagonistic activity of endophytes reduces pathogen load and contributes to overall plant health. We did not find any correlation between the source of bacteria isolation (roots or shoots) and their antifungal activity. There were four isolates from roots and four from shoots with antifungal activity against *F. culmorum* and *F. solani*, and four isolates from roots and six from shoots with antifungal activity against *R. solani*. The different number of isolates from roots and shoots with activity against *R. solani* is due to two active isolates (BRN1 and BRN3) being found both in roots and shoots. Higher percentages of endophytes with antifungal characteristics were observed in previous studies on *Chelidonium majus* L. [58] and *Hypericum perforatum*–associated bacteria [16],[32]. There is evidence that the physiological processes of endophytic bacteria residing inside plant tissue may be influenced by the biologically active components of medicinal plants [27],[39],[59]. Mehanni and Safwat [59] argued that endophytic bacteria may exhibit comparable biological activity and metabolite production to those of their hosts. The claim was validated by the research conducted by Koberl et al. [27] concerning endophytic bacteria extracted from the medicinal herbs *Solanum distichum, Matricaria chamomilla*, and *Calendula officinalis*, as well as endophytic bacteria isolated from *Hypericum perforatum*, which exhibited antifungal properties as their host. Furthermore, research revealed that fungal pathogens might be effectively suppressed without seriously harming the host by utilizing antagonistic characteristics of endophytic bacteria [60]–[62]. Endophytes associated with *Monarda citriodora*, for instance, demonstrated antagonistic action against *Fusarium oxysporum*, while *F. redolens* demonstrated potential for biocontrol [28].

The antagonism of endophytes against plant pathogens is mediated through several well-defined mechanisms such as the synthesis of siderophores, enzymes that break down fungal cell walls, and hydrogen cyanide (HCN) [63]–[65]. Chitinase, protease, glucanase, and lipase are the four tested enzymes that break down fungal cell walls. For example, chitinase can break down the essential component of the fungal cell wall, protease can break down fungal proteins, lipase can break down some lipids associated with the fungal cell wall, and β-1,3-glucanase can break down cell wall carbohydrates [16]. Of the fifteen bacterial strains, eleven produced at least one of these enzymes. Our findings on the antagonistic activity of endophytes against plant pathogens are well-supported by various studies. In our previous study, the bacterial strains *S. plymuthica* RR2-5-10 and *P. extremorientalis* TSAU20 were able to produce the cell wall–degrading enzyme protease and showed biological control of cucumber root rot caused by *Fusarium solani* [60]. Nineteen of the bacterial strains showed evidence of producing hydrogen cyanide (HCN), a process that also inhibits soil-borne pathogens [66]. According to Michelsen and Stougaar [67], isolates of *Pseudomonas fluorescens* that produced hydrogen cyanide (HCN) impeded *Rhizoctonia solani* and *Pythium aphanidermatum*'s hyphal development.

It is known that beneficial bacteria can produce phytohormones such as auxins (e.g., indole-3-acetic acid), gibberellins, and cytokinins, which promote plant growth and development. Eight of the fifteen bacterial strains we studied produced IAA and induced the growth of the fennel seedlings' roots or shoots. Several studies have documented the synthesis of indole-3-acetic acid (IAA) by endophytic bacteria linked to different medicinal plants, including *Thymus vulgaris, Majorana hortensis, Ocimum basilicum, Melissa officinalis, Marrubium vulgare, Solidago virgaurea*, *Melilotus officinalis*, and *Matricaria chamomilla* [68]. In pot trials, the endophytic bacteria isolated from *Cassia occidentalis* promoted mung bean plant growth by producing IAA [69]. Phytohormones play crucial roles in regulating plant growth and development processes such as cell elongation, division, and differentiation. The modest increases in fennel growth parameters observed in this study could be attributed to the endogenous production of such hormones by the endophytic bacteria, which might have influenced root and shoot development [70]–[73].

Ethylene regulates plant responses to abiotic stresses such as high salinity, extreme temperatures, and heavy metals. The enzyme ACC deaminase is produced by plant-associated bacteria and has the ability to reduce levels of the ethylene precursor, ACC (1-aminocyclopropane-1-carboxylic acid), within plant tissues. By lowering ACC levels, ACC deaminase effectively decreases ethylene production in plants [74]. Seven of the fifteen endophytic bacteria studied were capable of producing ACC deaminase. By reducing ethylene levels, ACC deaminase can help plants better tolerate these stresses. Although this study did not specifically measure stress parameters, the presence of endophytes might have contributed to a more robust stress response, allowing fennel plants to allocate resources more efficiently toward growth. In our previous study, the ACC deaminase-producing bacterial strains *P. putida* TSAU1 and *P. aureantiaca* TSAU22 stimulated the wheat root system in saline soils [47].

Eight out of fifteen bacterial strains produced siderophores. Microbial siderophores play an important role as determinants of biocontrol activity and influence the iron nutrition of plants [75],[76]. Seven out of fifteen bacterial strains possessed phosphate-solubilizing activity. Phosphate-solubilizing bacteria improve plants' phosphate nutrition by solubilizing insoluble phosphates in the soil and increasing the amount of phosphorus available for plants [77].

These traits, often exhibited by beneficial bacteria, can improve the nutrient availability in the rhizosphere, thereby promoting better growth and development of the plant. In the case of fennel, the observed increase in shoot and root length and dry weight suggests a potential improvement in nutrient uptake efficiency facilitated by the introduced endophytes. In this study, the introduction of endophytic bacteria into fennel seeds demonstrated positive, albeit modest, effects on the growth parameters of the plant.

Numerous papers have documented how endophyte inoculation improves plant growth. For example, Sudarshna and Sharma [78] reported that endophytic bacteria isolated from the medicinal plant *Trillium govanianum* increased plant growth and nutrient uptake of the plant under field conditions. The bacterial isolates demonstrated P solubilization activity and production of IAA, siderophore, and ACC deaminase. Similar results were obtained by Deepa et al. [79], whereas bacterial endophytes from *Pelargonium graveolens* demonstrated plant-beneficial traits and increased plant biomass and content of the essential oils geraniol and citronellol.

## 5. Conclusions

For the first time, endophytic bacteria from fennel (*Foeniculum vulgare* Mill.) samples taken from Uzbekistan's Ugam-Chatkal State Biosphere Reserve have been isolated, identified, and characterized in this work. Species belonging to *Bacillus, Pseudomonas, Brevibacterium, Enterobacter, Klebsiella*, and *Stenotrophomonas* were isolated and identified. In addition to demonstrating antifungal action against plant pathogenic fungi, the bacterial strains associated with fennel were found to be capable of synthesizing chitinase, protease, glucanase, lipase, HCN, siderophores, IAA, and ACC deaminase. According to our research, antimicrobial-rich medicinal plants may serve as a reservoir for microorganisms that exhibit antagonistic action against plant fungal pathogens, making them attractive options for the management of fungal diseases. They can also serve as an active part of biopreparation improving plant growth. These results also indicate that more investigation is required to determine how endophytic bacteria with particular plant growth promoting properties affect plant development and fungal disease control in field and pot studies. Further research should aim to optimize the use of endophytes to maximize their benefits and better understand their interactions with medicinal plants.
